# *Campylobacter fetus* Subspecies Contain Conserved Type IV Secretion Systems on Multiple Genomic Islands and Plasmids

**DOI:** 10.1371/journal.pone.0152832

**Published:** 2016-04-06

**Authors:** Linda van der Graaf–van Bloois, William G. Miller, Emma Yee, Gregor Gorkiewicz, Ken J. Forbes, Aldert L. Zomer, Jaap A. Wagenaar, Birgitta Duim

**Affiliations:** 1 Department of Infectious Diseases and Immunology, Faculty of Veterinary Medicine, Utrecht University, Utrecht, The Netherlands; 2 WHO Collaborating Centre for *Campylobacter* / OIE Reference Laboratory for Campylobacteriosis, Utrecht, The Netherlands; 3 Produce Safety and Microbiology Research Unit, Agricultural Research Service, U.S. Department of Agriculture, Albany, California, United States of America; 4 Institute of Pathology, Medical University of Graz, Graz, Austria; 5 School of Medicine and Dentistry, University of Aberdeen, Aberdeen, United Kingdom; 6 Central Veterinary Institute of Wageningen UR, Lelystad, The Netherlands; Indian Institute of Science, INDIA

## Abstract

The features contributing to differences in pathogenicity of the *Campylobacter fetus* subspecies are unknown. Putative factors involved in pathogenesis are located in genomic islands that encode a type IV secretion system (T4SS) and fic domain (filamentation induced by cyclic AMP) proteins, which may disrupt host cell processes. In the genomes of 27 *C*. *fetus* strains, three phylogenetically-different T4SS-encoding regions (T4SSs) were identified: one was located in both the chromosome and in extra-chromosomal plasmids; one was located exclusively in the chromosome; and one exclusively in extra-chromosomal plasmids. We observed that *C*. *fetus* strains can contain multiple T4SSs and that homologous T4SSs can be present both in chromosomal genomic islands (GI) and on plasmids in the *C*. *fetus* strains. The GIs of the chromosomally located T4SS differed mainly by the presence of *fic* genes, insertion sequence elements and phage-related or hypothetical proteins. Comparative analysis showed that T4SS sequences, inserted in the same locations, were conserved in the studied *C*. *fetus* genomes. Using phylogenetic analysis of the T4SSs, it was shown that *C*. *fetus* may have acquired the T4SS regions from other *Campylobacter* species by horizontal gene transfer. The identified T4SSs and *fic* genes were found in Cff and Cfv strains, although the presence of T4SSs and *fic* genes were significantly associated with Cfv strains. The T4SSs and *fic* genes could not be associated with S-layer serotypes or geographical origin of the strains.

## Introduction

*Campylobacter fetus* (*C*. *fetus*) contains currently three subspecies: *C*. *fetus* subsp. *fetus* (Cff), *C*. *fetus* subsp. *venerealis* (Cfv) and *C*. *fetus* subsp. *testudinum* (Cft), and the Cfv variant *C*. *fetus* subsp. *venerealis* biovar intermedius (Cfvi) [1,2]. Cff and Cfv are primarily associated with mammals [1,3], whereas Cft is associated with reptiles [2,4]. Cff and Cfv are highly related at the genome level [5,6], but are adapted to distinct hosts. *C*. *fetus* subsp. *fetus* can cause sporadic infections in humans, abortion in cattle and sheep and can be isolated from a variety of sites in different hosts [7]. *C*. *fetus* subsp. *venerealis* is restricted to the genital tract of cattle and is the causative agent of Bovine Genital Campylobacteriosis (BGC), a syndrome characterized by fertility problems in cattle [8].

A 57 kb genomic island encoding a type IV secretion system (T4SS) was identified in Cfv by Gorkiewicz *et*. *al*.[9]. This T4SS is analogous to the T4SS of *Agrobacterium tumefaciens*, and is considered to function as a type IVa class T4SS. In *A*. *tumefaciens*, the type IV translocation pilus is encoded by the *virB* operon that consists of eleven genes (*virB1-virB11*); translocation is also dependent on an additional gene *virD4*, which encodes the type IV coupling protein T4CP. The previously-identified genomic island of Cfv contains the *virB/virD4* T4SS, plasmid-related genes and two fic (filamentation induced by cAMP) domain-encoding genes [9]. The T4SS of Cfv has been shown to be functional and supports intra- and interspecies conjugative DNA transfer [10]. The fic domain proteins have critical roles in multiple cellular processes, including disrupting the host cell processes that are important to pathogen survival and replication, after transmission into eukaryotic cells [11]. It was hypothesized that this genomic island is responsible for the pathogenicity and clinical symptoms manifested during Cfv infections [10].

Other features responsible for the pathogenicity of *C*. *fetus* strains are the surface layer proteins (SLPs) that cover *C*. *fetus* cells [12–15]. The *C*. *fetus* SLPs undergo antigenic variation and protect the cell against the host immune system. The mammalian *C*. *fetus* strains can be serotyped into two major groups, serotype A or serotype B [16]. As both the S-layer proteins and T4SS regions are suggested to have a role in the pathogenicity of *C*. *fetus*, it might be possible that these features have a synergistic role in immune escape.

In the first description of a *C*. *fetus* genomic island harboring a T4SS, it was concluded that this genomic island was specific for *C*. *fetus* subsp. *venerealis* [9]. From the recently published *C*. *fetus* genomes [17–19], it has become clear that the genome of Cff strain 04/554 contains a T4SS on a megaplasmid and that some *C*. *fetus* strains can even harbor multiple T4SSs. The genome of Cfv strain 84–112 harbors four T4SSs; two genomic islands contain a T4SS and two T4SSs were located in an extra-chromosomal element [17]. It is unknown if *C*. *fetus* commonly harbors multiple T4SS-encoding regions and how dispersed the different T4SSs are among *C*. *fetus* strains and the *C*. *fetus* subspecies.

In this study, we examined the diversity of T4SS-encoding regions in 27 *C*. *fetus* strains using comparative genomics, and identified the location and composition of all T4SS encoding regions and their phylogeny. Furthermore, we studied whether the presence of specific T4SSs and *fic* genes could be associated with the *C*. *fetus* subspecies, their pathogenicity, the S-layer serotypes and geographic origin of the strains. Phylogenetic analysis with T4SSs of other *Campylobacter* species suggested that the *C*. *fetus* T4SS regions did not evolve from the same ancestor, but were acquired from different donors.

## Materials and Methods

### Bacterial strains

In this study, 27 *C*. *fetus* strains from different countries and sources were analysed (Table 1). The phenotypic and genotypic characteristics of the strains were described previously [19].

**Table 1:** Characterization and prevalence of T4SS regions in *C*. *fetus* strains.

				Identification															
Strain	Accession number	Country	Source	Phenotypic ID	Genotypic ID	Sap	Pfam*	Pfam*	T4SS (sub)clusters**										
						serotype	(VirD4)	(Fic)	1						2		3		
				[19]	[19]	[19]			A	B	C	D	E	F	A	B	A	B	C
BT 10/98	LRAL00000000	UK	Ovine	Cff	Cff	A	-	-	-	-	-	-	-	-	-	-	-	-	-
B0097	ERR419623	UK	Bovine (faeces)	Cff	Cff	A	-	-	-	-	-	-	-	-	-	-	-	-	-
110800-21-2	LSZN00000000	NL	Bovine (bull)	Cff	Cff	A	1	2	+	-	-	-	-	-	-	-	-	-	-
98/v445	LMBH00000000	UK	Bovine (bull)	Cff	Cff	B	4	4	+	-	-	-	-	-	-	+	-	+	+
B0066	ERR419610	UK	Bovine (faeces)	Cff	Cff	B	-	-	-	-	-	-	-	-	-	-	-	-	-
04/554	CP008808-008809	AR	Bovine (foetus)	Cff	Cff	B	1	4	-	-	-	-	-	-	-	-	-	-	+
B0167	ERR460866	UK	Bovine (faeces)	Cff	Cff	B	-	4	-	-	-	-	-	-	-	-	-	-	-
82–40	CP000487	US	Human (blood)	Cff	Cff	A	-	-	-	-	-	-	-	-	-	-	-	-	-
B0131	ERR419639	UK	Bovine (faeces)	Cff	Cff	A	-	-	-	-	-	-	-	-	-	-	-	-	-
03/293	CP0006999-007002	AR	Bovine (foetus)	Cff	Cfvi	A	3	3	+	-	+	-	-	-	+	-	-	-	-
ADRI 1362	LREX00000000	AR	Bovine	Cff	Cfvi	A	4	3	-	-	+	+	-	-	+	-	-	-	+
Zaf 65	LREY00000000	SA	Bovine	Cff	Cfvi	A	3	6	-	+	-	-	+	-	-	-	-	+	-
01/165	CP014568-014570	AR	Bovine (mucus)	Cfvi	Cfvi	A	3	2	-	-	-	-	-	+	+	-	+	-	-
02/298	LRVK00000000	AR	Bovine (foetus)	Cfvi	Cfvi	A	4	4	+	-	+	-	-	-	+	-	-	-	+
03/596	LRAM00000000	AR	Bovine (foetus)	Cfvi	Cfvi	A	2	3	-	-	-	-	-	-	+	-	-	-	+
92/203	LRVL00000000	AR	Bovine (placenta)	Cfvi	Cfvi	A	3	4	+	-	-	-	-	+	+	-	-	-	-
97/532	LRER00000000	AR	Bovine (mucus)	Cfvi	Cfvi	A	3	8	-	-	-	-	-	+	+	-	-	-	+
98/25	LRES00000000	AR	Bovine (foetus)	Cfvi	Cfvi	A	4	4	-	-	+	-	-	-	+	-	+	-	+
WBT 011/09	LMBI00000000	UK	unknown	Cfvi	Cfvi	A	5	4	+	+	-	-	-	+	+	+	-	-	-
Zaf 3	LREZ00000000	SA	Bovine (foetus)	Cfvi	Cfvi	A	2	6	-	-	-	-	+	-	-	-	-	-	+
ADRI 513	LRFA00000000	AU	unknown	Cfvi	Cfv	A	4	7	-	-	-	-	+	-	-	-	+	+	+
CCUG 33872	LREU00000000	CZ	unknown	Cfvi	Cfv	A	4	8	-	-	-	-	+	-	-	-	+	+	+
84–112	HG004426-004427	US	Bovine	Cfv	Cfv	A	4	4	+	-	+	-	-	-	+	-	-	-	+
97/608	CP008810-008812	AR	Bovine (placenta)	Cfv	Cfv	A	4	3	+	-	+	-	-	-	+	-	-	-	+
B10	LRET00000000	US	Bovine	Cfv	Cfv	A	3	7	-	-	-	-	+	-	+	-	-	-	+
CCUG 33900	LREV00000000	FR	Bovine (abortion)	Cfv	Cfv	A	2	4	-	-	-	-	-	-	+	-	-	-	+
LMG 6570	LREW00000000	BE	Bovine	Cfv	Cfv	A	3	3	+	-	-	-	-	-	+	-	-	-	+

* Numbers refer to present proteins.

**Fig 1:**
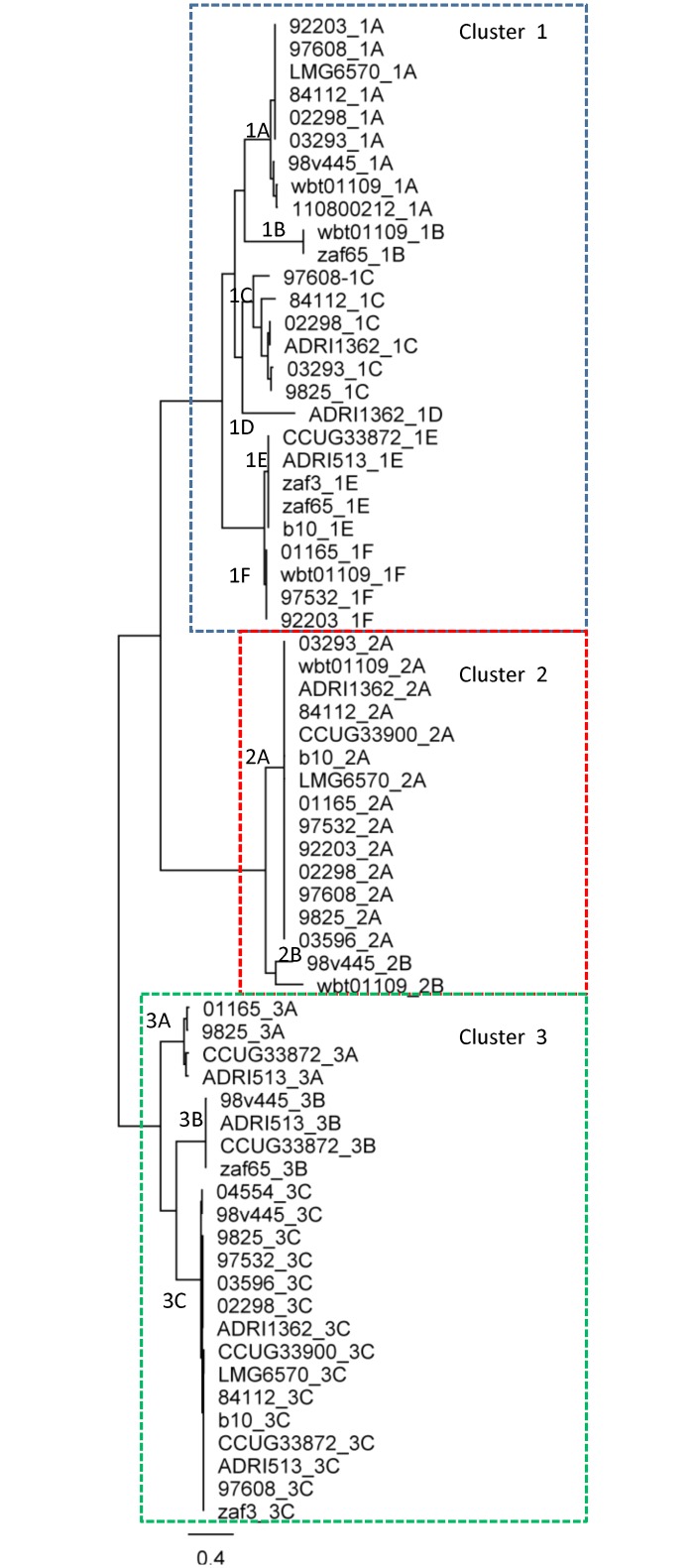
Phylogenetic analysis of *virD4* genes. The scale bar represents the mean number of nucleotide substitutions per site. Related *virD4* genes are indicated in boxes.

** Classification according to Fig 1. +; region is present,—; region is absent. Country code: AR, Argentina; AU, Australia; BE, Belgium; CZ, Czech Republic; FR, France, NL, Netherlands; SA, South Africa; UK, United Kingdom; US, United States. Abbreviations: Cff, Campylobacter fetus fetus; Cfv, Campylobacter fetus venerealis; Cfvi, Campylobacter fetus venerealis biovar intermedius.

### Whole genome sequencing

The *C*. *fetus* strains (except strains B0066, B0097, B0131 and B0167) were sequenced using a Roche 454 GS-FLX+ Genome sequencer with Titanium chemistry. Roche 454 reads were assembled into contigs using the Newbler Assembler (version 2.6). The remaining four Cff strains (B0066, B0097, B0131 and B0167) from the UK were sequenced according to the following procedure; the isolation of genomic DNA for whole genome sequencing (WGS) used the Promega Wizard Genomic DNA Purification Kit. All of the DNA samples went through a genomic library prep which is similar to the Illumina Truseq protocol, but which was developed at the Sanger Institute. The libraries were sequenced on Illumina HiSeq 2000 analysers on 100bp paired end runs. The paired read files were de novo assembled using the Velvet assembler in an established pipeline at the Sanger Institute.

To span repeat regions, four *C*. *fetus* genomes (04/554, 97/608, 03/293 and 01/165) were sequenced with a PacBio RS sequencer (Keygene N.V., Wageningen, the Netherlands). PacBio RS reads were assembled into contigs using Quiver (Pacific Biosciences, CA, USA) and the base calls were validated with Illumina MiSeq reads.

The reference genomes of strain 82–40 genome (Genbank accession number CP000487), strain 84–112 (Genbank accession numbers HG004426-HG004427), strain 03/293 (Genbank accession numbers CP0006999-CP007002), 04/554 (Genbank accession numbers CP008808-CP008809) and strain 97/608 (Genbank accession numbers CP008810-CP008812) were used to determine the exact locations of T4SS encoding regions. The sequences of strains B0066, B0097, B0131 and B0167 (with accession numbers starting with ERR) are available from the Wellcome Trust Sanger Institute, and the remaining sequences are available from NCBI Genbank (Table 1).

### Pfam search and phylogenetic analysis of *virD4* and *fic* genes

From the Roche 454 and Illumina contigs, predicted gene nucleotide and protein sequences were generated using GeneMark.hmm version 3.25 [20]. The predicted protein sequences were used in a Pfam search (version 27.0; Pfam.xfam.org) [21] to identify the matching Pfam families of the proteins. For each genome, proteins that matched with Pfam family T4SS-DNA_transf (PF02534.9) and Fic (PF02661.13) were selected. Genes matching Pfam family T4SS-DNA_transf were annotated as *virD4*.

A Fisher’s exact test was used to calculate the two-tail probability value (p) between the presence of *virD4* and *fic* genes versus the different serotypes and subspecies of the strains.

Phylogenetic analysis of the *virD4* and *fic* genes was performed by alignment of these genes with MUSCLE [22] and building a maximum likelihood tree using RAxML (v7.2.8) under the GTRCAT model. Four *fic* genes were included in the phylogenetic analysis as reference: *fic1* (Genbank accession number ACS15152), *fic2* (Genbank accession number ACA64462), *fic3* (Genbank accession number CDF65920) and *fic4* (Genbank accession number CDF65967). The positions of the *fic* genes of the in this study analysed sequences are listed in S1 Table.

### Analysis of T4SS encoding regions

The WGS contigs containing the *virD4* gene sequences, as defined above, were sorted out for each genome. The location of each T4SS region was identified by tracing the core genes adjacent to the T4SS genes on the contigs. Using the reference genomes of strains 84–112, 01/165, 04/554, BRIG alignments were created with a 70% upper identity threshold and 50% lower identity threshold [23]. Phylogenetic analysis of the *virB9* genes and complete T4SSs was performed as described for the *virD4* and *fic* genes.

### Comparison with T4SS proteins from other *Campylobacter* species

To calculate the homology of T4SS proteins with proteins from other *Campylobacter* species, a BLASTP comparison to the proteins in the NCBI non-redundant database was performed. The phylogenetic comparison of the *C*. *fetus* VirD4 proteins with proteins from other *Campylobacter* species was performed by alignment of these genes with MUSCLE [22] and building a maximum likelihood tree using RAxML (v7.2.8) under the GTR model with gamma correction. For *C*. *fetus*, one VirD4 protein from each phylogenetic cluster was included. The VirD4 protein sequences of other *Campylobacter* species were obtained from GenBank submissions.

## Results

### Analysis of the *fic* and *virD4* genes and T4SS encoding regions

For each strain, the number of genes that matched with Pfam families T4SS-DNA_transf (*virD4*) and Fic (*fic*) are listed in Table 1.

Phylogenetic analysis of the *fic-*encoding genes demonstrated that the *fic* genes are highly diverse and can be divided into multiple clusters (S1 Fig). Almost all analysed *C*. *fetus* strains contained multiple *fic* genes, except strains 82–40, BT10/98, B0066, B0097 and B0131. These strains also lacked *virB* and *virD4* genes. Cff strains 04/554 and B0167 contained four *fic* genes in a genomic island, as shown with the BRIG analysis in S2 Fig. The presence of the *fic* genes is significantly associated with *C*. *fetus* subsp. *venerealis* (p = 0.001). The presence of *fic* genes was not significantly associated with the geographical origin and serotypes of the strains (p = 1.0).

The identified *virD4* genes were classified into three main phylogenetic clusters (Fig 1: clusters 1–3). These clusters were divided further into multiple sub-clusters, designated 1A-1F, 2A-2B and 3A-3C (Fig 1). The *virD4* genes of clusters 1 and 2 are all located within T4SS regions consisting of *virB2-virB11* genes. The *virD4* genes of cluster 3 are located on plasmids, in T4SS regions encoding *tra* and *trb* conjugative transfer genes. The *tra/trb* regions of cluster 3 are more diverse in composition than the sequences of the *virB/virD4* regions (data not shown). The highly diverse *tra/trb* regions of cluster 3 were excluded from assemblies of the T4SS regions and their adjacent genes.

**Fig 2:**
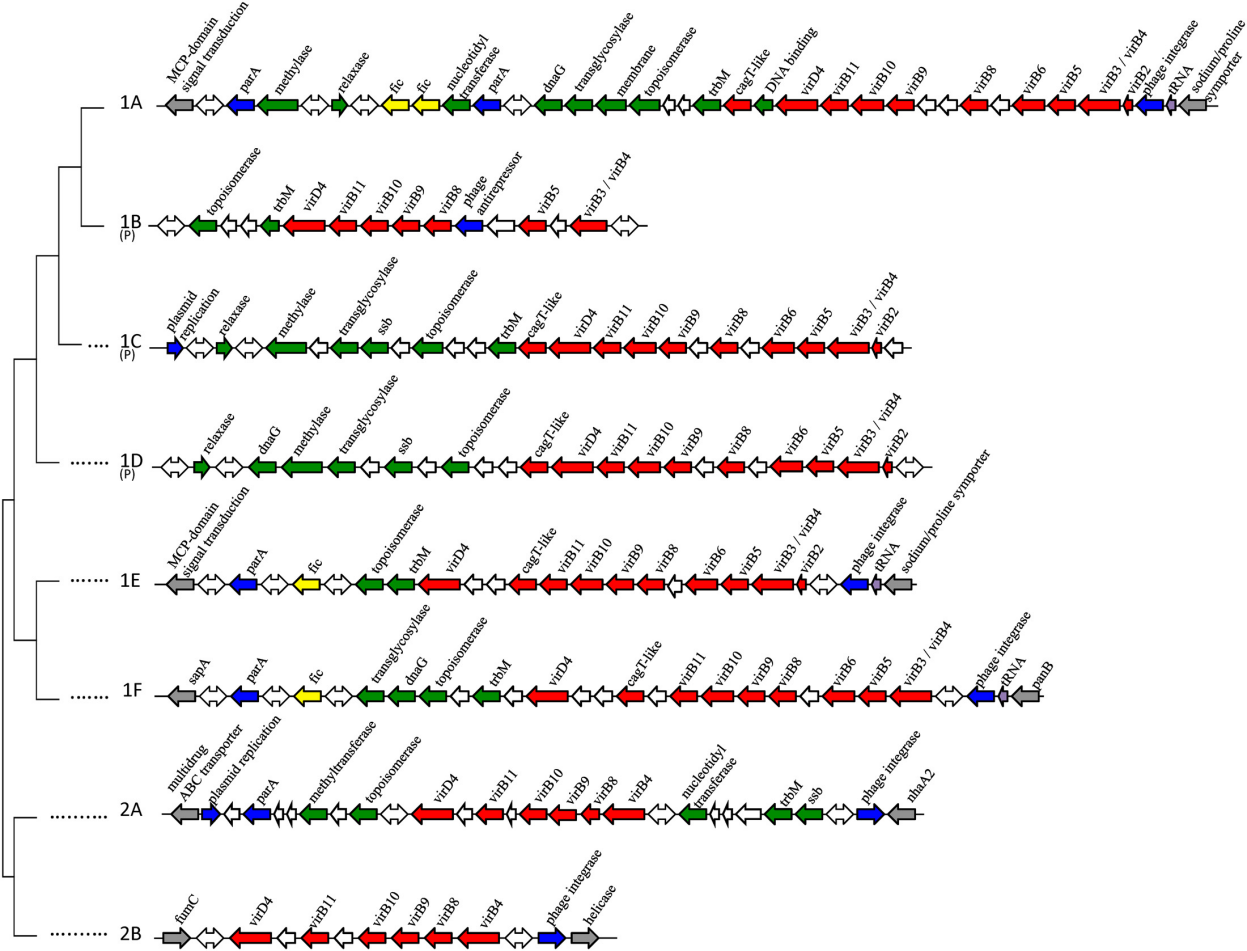
Schematic representation of T4SS regions in genomic islands and plasmids. The T4SS regions and their adjacent genes are listed according to the phylogenetic clustering as shown in Fig 1. The T4SS regions in genomic islands or plasmids (P) contain: *vir*/*cag* genes (red), phage integrases and other phage-related genes (blue), genes of plasmid origin (green), tRNA genes (purple), chromosomal integration sites (grey) and genes of unknown function (white). Double-sided arrows indicate genes of unknown function in varying orientations. The genomic islands of cluster 2 are in the chromosomes located in the opposite orientation as shown in Fig 2.

The T4SS regions of cluster 1 are located either in a genomic island (GI) or on a plasmid, whereas the T4SS regions of cluster 2 are located only in a GI. The adjacent genes of the T4SS regions in the GIs and the plasmids were identified (Fig 2). This enabled the assembly of the T4SS-encoding region, which showed extensive diversity in gene content. The typical features of the T4SS regions and their accompanying genes are shown in Table 2. Additionally, through alignment with the reference genomes of strains 84–112 and 01/165, the location of the chromosomal T4SS regions 1A, 1F and 2A in the genomes is shown in S2 Fig.

**Table 2:** Typical features of C. fetus T4SS encoding regions.

Cluster		Location	Transfer-associated genes				*fic* genes (n)	T4SS genes		T4SS with highest homology
			*parA*	*dnaG*	*trbM*	*cagT*-like		present	absent	
	A	GI	+	+	+	+	2	*virB2-virD4*		*C*. *hominis*,
**1**	B	plasmid	-	-	+	-	-	*virB3-virD4*	*virB2*, *virB6*	*C*. *ureolyticus*
	C	plasmid	-	-	+	+	-	*virB2-virD4*		
	D	plasmid	-	+	-	+	-	*virB2-virD4*		
	E	GI	+	-	+	+	1	*virB2-virD4*		
	F	GI	+	+	+	+	1	*virB3-virD4*	*virB2*	
**2**	A	GI	+	-	+	-	-	*virB4-virD4*	*virB2*, *virB3*, *virB5*, *virB6*	*C*. *concisus*,
										*C*. *showae*, *C*. *rectus*
	B	GI	-	-	-	-	-	*virB4-virD4*	*virB2*, *virB3*, *virB5*, *virB6*	
**3**		plasmid						*tra*, *trb*		*C*. *coli* plasmid

GI; genomic island, +; gene is present,—; gene is absent

Phylogenetic clustering of the *virD4* genes is similar to the clustering of both the *virB9* genes and the complete T4SSs (data not shown), demonstrating not only conservation of the sequences of *virD4* gene sequences, but also conservation of the entire T4SS-encoding regions within *C*. *fetus*.

### Location and gene content of the cluster 1 T4SS regions

The T4SS regions of cluster 1 are located in genomic islands (regions 1A, 1E and 1F) as well as on plasmids (regions 1B, 1C and 1D) (Fig 2). The T4SS region 1A is the most studied T4SS of *C*. *fetus* [9,10,17], and is located in the most complex genomic island of cluster 1 (Fig 2). The T4SS regions 1A and 1E are phylogenetically positioned in different sub-clusters (Fig 1), but the genomic islands of these T4SS regions are integrated in the same location in the chromosome, between an MCP-domain signal transduction protein (GenBank accession no. YP_892387.1) and a sodium/proline symporter (GenBank accession no. YP_892386.1). The plasmid-associated T4SS region 1B is smaller and is lacking the *virB6* gene, possibly due to the insertion of the phage anti-repressor gene (Fig 2). T4SS region 1F was found in two strains and was located in both strains in a genomic island in the *sap* locus, between a *sapA* homolog and *panB* (GenBank accession no. YP_891665.1) (Fig 2).

The GI of T4SS region 1A contains two *fic* genes, whose products are possibly secreted by the T4SS [10]. The genomic islands of the other chromosomally-located T4SS regions 1E and 1F contain a single *fic* gene. No *fic* genes were found adjacent to the plasmid-associated T4SS regions 1B, 1C and 1D (Table 2, Fig 2).

Adjacent to the T4SS regions, multiple transfer-associated genes are found, e.g. *parA*, *dnaG*, *trbM* and a *cagT*-like gene (Table 2). Beyond the transfer-associated genes, the T4SS-adjacent regions can contain genes encoding a nickase, an *Eco*RI methyltransferase, a helicase, phage-associated genes and genes encoding hypothetical proteins (Fig 2).

**Fig 3:**
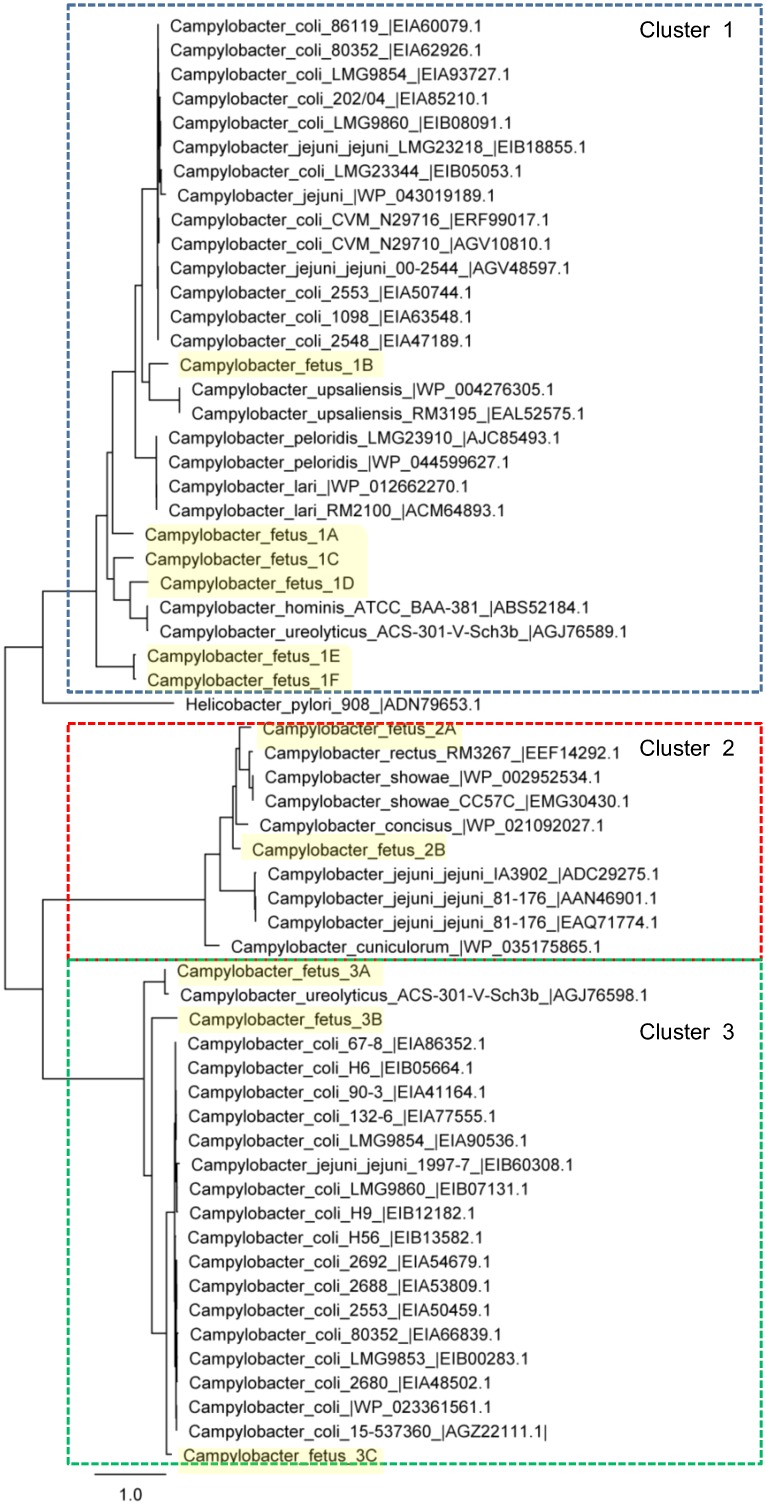
Phylogenetic analysis of virD4 proteins of different *Campylobacter* species. The scale bar represents branch length (number of amino acid substitutions/100 residues). Related VirD4 proteins are indicated in boxes.

The VirD4 proteins of cluster 1 were similar to the VirD4 proteins of multiple other *Campylobacter* species. Most of the VirD4 proteins encoded by other *Campylobacter* species are of plasmid origin (e.g., *C*. *upsaliensis* EAL52575.1, *C*. *peloridis* AJC85493.1, *C*. *lari* ACM64893.1, *C*. *coli* and *C*. *jejuni* VirD4 proteins). The genes encoding the VirD4 proteins of *C*. *hominis* and *C*. *ureolyticus* were located on their respective chromosomes; these VirD4 proteins are positioned close to the two plasmid-encoded VirD4 proteins of *C*. *fetus*, 1C and 1D (Fig 3).

### Location and gene content of the cluster 2 T4SS regions

The T4SS regions of cluster 2 are exclusively found in *C*. *fetus* genomic islands. The GI containing T4SS region 2A is inserted between genes encoding a multidrug resistance ABC transporter (GenBank Accession no. YP_892875.1) and *nhaA2* (GenBank Accession no. YP_892871.1). The GI of T4SS region 2B is inserted between genes encoding the class II fumarate hydratase FumC (GenBank Accession no. YP_892133.1) and a UvrD/rep helicase (GenBank Accession no. YP_892129.1) (Fig 2).

The T4SS regions 2A and 2B are lacking *virB2*, *virB3*, *virB5* and *virB6*, and both genomic islands do not contain *fic* genes. The GI of T4SS region 2B contains only the T4SS genes. The GI of T4SS region 2A contains the transfer-associated genes *parA* and *trbM* (Table 2). Furthermore, the GI of T4SS region 2A encodes a nucleotidyltransferase, a topoisomerase and an *Eco*RI methyltransferase, like the GIs of cluster 1 (Fig 2).

Comparison with the T4SS proteins of other *Campylobacter* species in the NCBI non-redundant database showed that the *C*. *fetus* VirD4 protein of cluster 2 shared 78% homology with the VirD4 proteins of *C*. *concisus* (GenBank accession no. WP_021092027.1) and *C*. *rectus* (Genbank accession no. WP_039888059.1) and 79% homology with the VirD4 protein of *C*. *showae* (Genbank accession no. WP_002952534.1). The gene contents of the T4SSs of cluster 2 are similar to the *vir* operon of *C*. *showae* and *C*. *rectus* [24], consisting of *virB4*, *virB8*, *virB9*, *virB10*, *virB11* and *virD4*. Furthermore, the GIs of T4SS region 2A contain, similar to the C. *showae* GI T4SS region, nucleotidyltransferase-, topoisomerase- and *Eco*RI methyltransferase- encoding genes (Fig 2: T4SS 2A). The *C*. *fetus* VirD4 proteins of cluster 2 are chromosomally-encoded, but are phylogenetically positioned close to plasmid-encoded VirD4 proteins of three *C*. *jejuni* strains (Genbank accession nos. EAQ71774.1, ADC29275.1 and AAN46901.1) (Fig 3).

### Gene content of the T4SS regions of cluster 3

The *virD4* genes of cluster 3 were located in a T4SS region encoding *tra* and *trb* conjugative transfer genes. As with the plasmid-encoded *virB/virD* region, the *tra/trb* gene cluster is also found in the octopine-type Ti plasmids of *Agrobacterium tumefaciens* [25]. In *C*. *fetus*, this T4SS is exclusively located on plasmids and was not identified in the chromosomes of the analysed strains. The *tra/trb* T4SS encoding region is located in the extra-chromosomal element ICE_84112 of Cfv strain 84–112 [17] and on the megaplasmids of strain Cff 04/554 and Cfv 97/608 [19]. The *tra/trb* gene clusters were highly diverse in gene content. Furthermore, in both closed Cfv genomes 84–112 and 97/608, this T4SS region is disrupted by several insertion sequence elements. In strains 04/554 and 97/608, both megaplasmids with the *tra/trb* region contain one adjacent *fic* gene. The ICE of strain Cfv 84–112 contains two *fic* genes, but this ICE contains also a *virB/virD4* T4SS, and it is not known if the *fic* genes are linked to the *tra/trb* T4SS or the *virB/virD4* T4SS of this ICE or to both T4SS regions.

The proteins of the *tra/trb* T4SS share a high sequence identity (>90%) with a conjugal transfer locus in a *C*. *coli* plasmid [26]. The *C*. *fetus* VirD4 proteins 3A-3C clustered with a large group of mainly plasmid-encoded VirD4 proteins present in *C*. *coli* and *C*. *jejuni* (Fig 3).

### Distribution of T4SS regions in *C*. *fetus* subspecies and association with pathogenicity, geographic origin and S-layer

Of the 27 analysed *C*. *fetus* strains, 21 strains contained a T4SS (Table 1). The T4SS regions of cluster 1 were identified in 18 strains, and the T4SS regions of cluster 2 in 15 strains. The majority of strains (n = 17) contain a *virD4* gene of the *trb/tra* T4SS region of cluster 3. It was common for strains to harbor multiple T4SSs (Table 1). The identified T4SSs were found in Cff and Cfv strains; Cff strain 98/v445 contains four T4SS regions and a single T4SS region was found in Cff strains 110800-21-2 and 04/554, showing that the T4SS regions are not *C*. *fetus* subsp. *venerealis* specific. However, the Fisher’s exact test showed that the presence of VirD4 proteins is significantly associated with *C*. *fetus* subspecies *venerealis* (p = 0.003).

From the set of 27 strains, nine strains were isolated from bovine abortions (strains 04/554, 03/293, 02/298, 03/596, 92/203, 98/25, Zaf3, 97/608 and CCUG 33900). Most of these strains (7 of the 9 strains; except strains 03/293 and 92/203) contained the *tra/trb* T4SS region 3C and most of these strains (8 of the 9 strains, except strain 04/554) contained one or more of the non-chromosomally-located T4SS regions 1B, 1C and 1D, showing that all *C*. *fetus* strains isolated from abortions contained at least one T4SS. Since the clinical data of the remaining 18 strains was not available, it was not possible to calculate if there is a significance association between the T4SSs and pathogenicity of the strains.

The strains used in this study were obtained from different countries and the T4SSs were distributed between strains from different countries, showing that the presence of a specific T4SS in *C*. *fetus* strains does not correlate with geographic origin.

The presence of T4SS regions was compared with the S-layer (Sap) serotypes of the *C*. *fetus* strains (Table 1). Cff strains of both Sap serotypes A and B contain the T4SS region of cluster 1. Cff strain 98/v445 of serotype B contained the T4SS region of cluster 2 as well as two *virD4* genes of the *trb/tra* T4SS region. These *trb/tra* T4SSs were absent in Cff serotype A strains, but are present in many Cfv serotype A strains. The presence of the *virD4* genes is not significantly associated with the serotypes of the *C*. *fetus* strains (p = 0.20).

## Discussion

*Campylobacter fetus* subspecies *fetus* and *C*. *fetus* subspecies *venerealis* are genetically highly related, but show a different pathogenicity and host adaptation. *C*. *fetus* subsp. *venerealis* (including Cfv biovar intermedius) is associated with Bovine Genital Campylobacteriosis and is restricted to the genital tract of cattle. *C*. *fetus* subsp. *fetus* is associated with sporadic abortions in cattle and has a broader host range possibly because of its ability to survive in the gastro-intestinal tract. What pathogenicity motifs could affect the different pathogenesis during the infection process of Cfv, Cfvi and Cff is unknown. This study demonstrates that multiple T4SSs, which have been suggested to be involved in the pathogenicity of *C*. *fetus* strains, are present in both subspecies and that the composition of the T4SS-encoding regions is highly diverse.

We have identified the T4SS-encoding regions by searching the genomes with a Pfam search for *virD4* genes, as it has been shown before that conjugative transfer systems can be found just by searching for known sequences, like relaxases, T4CPs (*virD4*) and *virB4* genes [27]. With this approach, we were able to identify three phylogenetically different T4SS regions in *C*. *fetus* strains.

### i. Subspecies specificity of the T4SSs and association with pathogenicity

The genomic island containing T4SS region 1A has been described as Cfv-specific with a prevalence of 76% in Cfv and complete absence in Cff strains [9]. Our study showed that Cff strains can harbour the complete *virB/virD* T4SS region 1A (Cff strains 110800-21-2 and 98/v445), as well as the plasmid encoded *tra/trb* T4SS region of cluster 3 (Cff strains 04/554 and 98/v445), confirming that the T4SS encoding regions are not Cfv-specific [28].

It has been suggested that genomic island genes could be used as specific targets to detect Cfv [29]. In this study, we demonstrated that T4SS genes are present in strains from both subspecies and that no T4SS class is subspecies-specific, confirming that subspecies identification cannot be accomplished using assays that detect T4SS genes.

Nine strains were isolated from bovine abortions; two phenotypic Cff strains and seven Cfv/Cfvi strains. These strains contained the *tra/trb* T4SS region 3C (except strains 03/293 and 92/203) and contained one or more of the non-chromosomally-located T4SS regions 1B, 1C and 1D (except strain 04/554). This suggests that the T4SSs have a potential role in the pathogenicity of the *C*. *fetus* strains and this is independent of the *C*. *fetus* subspecies. In this study, we were not able to calculate the significance of the association between the presence of a specific T4SS region to the pathogenicity of the *C*. *fetus* strains, because detailed information on the clinic and epidemiology of most of the strains was not available.

Since the S-layer proteins play an important role in the pathogenesis of *C*. *fetus* infections [12–15], it was studied if the serotypes of the *C*. *fetus* strains are associated with a T4SS region. The Fisher’s exact test showed no significant association of the serotypes and T4SSs of the strains, but one should take into account that only four strains with serotype B were included in this study, making it not possible to determine the association of serotypes and pathogenicity of the *C*. *fetus* strains.

### ii. Functionality of the T4SSs

*C*. *fetus* strains can harbour multiple T4SS-encoding regions. The T4SS region of cluster 1 was present in 18 of the 27 *C*. *fetus* strains. In 16 of these strains, a plasmid encoding a *tra/trb* T4SS region was also present, suggesting that multiple formats for conjugational transfer are present within *C*. *fetus*. The finding that Cff strains 82–40, BT 10/98, B0066, B0097 and B0131 lack any T4SS-encoding genes confirms that the T4SS is not essential for the *C*. *fetus* life cycle outside the bovine genital tract. All strains that were isolated from the bovine genital tract contained at least one T4SS.

Within the analysed T4SS regions, all *virB* and *virD4* genes are oriented in the same direction, suggesting an operon structure. Furthermore, both genomic islands of T4SS region 1A and 2A contain a nucleotidyltransferase, and adjacent to T4SS 1A, 1C and 1D a relaxase is found. The presence of these genes suggests a nucleic acid transport function of the T4SSs, as described for the T4SSs of *C*. *showae* and *C*. *curvus* [24].

The T4SS genes and composition of cluster 2 are highly homologous with the T4SSs found in *C*. *showae* and *C*. *rectus* [24]. In *C*. *fetus*, it is not demonstrated that this T4SS is functional [17] and it is unknown if this T4SS mediates conjugative DNA transfer between *C*. *fetus* strains.

An extensively studied virulence locus in *Helicobacter pylori* is the *cag* pathogenicity island (*cag*PAI) encoding a T4SS. The presence of a *cag*PAI discriminates the highly virulent *cag*PAI-positive *H*. *pylori* strains from the less virulent *cag*PAI-negative *H*. *pylori* strains [30]. In *H*. *pylori*, CagA is translocated into the cytoplasm of an infected cell by the T4SS, where it modulates the host immune system [31]. In *C*. *fetus*, it was hypothesized that the Fic proteins are translocated by the T4SSs, although the secretion of these proteins could not be proved [10]. The translocation of bacterial Fic proteins to the eukaryotic host affects important pathogen recognition processes in the host cell important for survival and replication [10]. Interestingly however, in this study, most of the Fic motif proteins were not located on a contig containing a T4SS gene cluster because of contig breaks caused by repetitive sequences. Therefore, we were not able to link the Fic proteins to the T4SS of the analyzed regions. Furthermore, Fic-domain containing proteins might be found in any integrated element [17] and thus their presence or absence is not always related to a T4SS.

### iii. Evolution and transfer of T4SSs

An interesting finding was the sequence conservation of the T4SS encoding genes in different *C*. *fetus* strains. Genomic islands are commonly acquired by horizontal gene transfer, followed by island evolution via genetic rearrangements, gene loss, mutations or acquisition of other mobile genetic elements [25]. The genomic islands containing the T4SS regions are inserted at different chromosomal locations, and gene loss and rearrangements are observed in the GIs of different strains, but the T4SS sequences are conserved in the respective *C*. *fetus* strains. This indicates an evolutionary relationship of the T4SS sequences, but also that T4SS sequences may be conserved to facilitate a functional conjugation system.

Multiple transfer-associated genes are found adjacent to the T4SS regions of cluster 1 and 2 (Table 2). The presence of these transfer-associated genes suggests a plasmid origin for these regions.

The T4SS regions 1B-1D are non-chromosomally located in megaplasmids or ICEs. These regions contained T4SS proteins that were highly homologous to those present in the chromosomally-located T4SS regions 1A, 1E and 1F. ICEs and plasmids can be transferred between cells using a T4SS [32]. The high homology of the chromosomally- and plasmid located T4SSs could also indicate that *C*. *fetus* strains contain a gene shuffling mechanism, with which plasmids might pick up either chromosomal genes or integrate sequence modules from foreign plasmids, as described for *H*. *pylori* [33].

Phylogenetic analysis of the VirD4 proteins showed that the *C*. *fetus* VirD4 proteins and the VirD4 proteins of different *Campylobacter* species form three clusters (Fig 3). This suggests that these *C*. *fetus* T4SS regions did not evolve from the same ancestor, but were acquired from different donors either by plasmid transfer or conjugational recombination to the *C*. *fetus* chromosomes.

## Conclusions

Overall, our study showed that *C*. *fetus* strains contain at least three distinct regions, wherein T4SSs could be located in a genomic island, on plasmids and both chromosomally as well as in extra-chromosomal elements. The presence or absence of T4SS is not related to the S-layer serotype or to the geographic origin of strains, but it is shown that the presence of *virD4* and *fic* genes is significantly associated with *C*. *fetus* subsp. *venerealis*. Furthermore, it is suggested that the pathogenicity of the *C*. *fetus* strains are not congruent with the *C*. *fetus* subspecies classification. Phylogenetic analysis of T4SS-encoding regions showed that the gene content of these regions is conserved in all the analysed *C*. *fetus* strains and showed that the T4SSs were most likely not acquired from a single ancestor but from different donors.

## Supporting Information

S1 Fig Phylogenetic analysis of Fic-encoding sequences. The scale bar represents the mean number of nucleotide substitutions per site.
(TIF)

S2 Fig BRIG alignment of chromosomally-located T4SS regions 1A, 1E and 2A, and *fic* genes. Shown are the locations of the chromosomal T4SS regions 1A, 1E and 2A in the reference genomes, using strain 84–112 or strain 01/165 as reference. Strain 04/554 is used as reference to show the location of a genomic island with four *fic-*encoding sequences.
(TIF)

S1 Table Genome positions of *fic* encodinggenes. (XLSX)
