# Supplementary figures and images for: Campylobacter fetus Subspecies Contain Conserved Type IV Secretion Systems on Multiple Genomic Islands and Plasmids
Source: PLoS One. 2016 Apr 6;11(4):e0152832. doi: 10.1371/journal.pone.0152832 (PMC4822827; doi:10.1371/journal.pone.0152832)

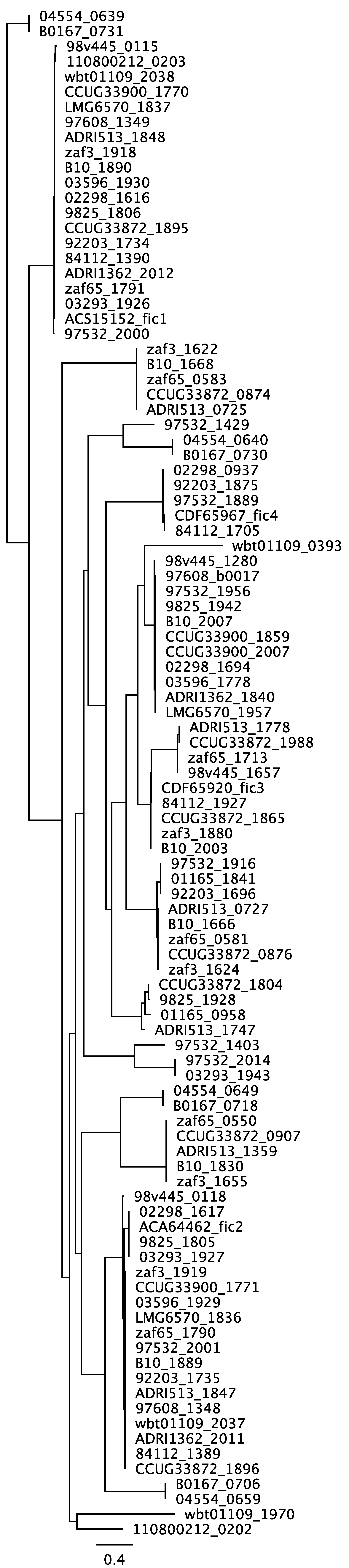

Supplement: S1 Fig — The scale bar represents the mean number of nucleotide substitutions per site. (TIF) [file pone.0152832.s001.tif]

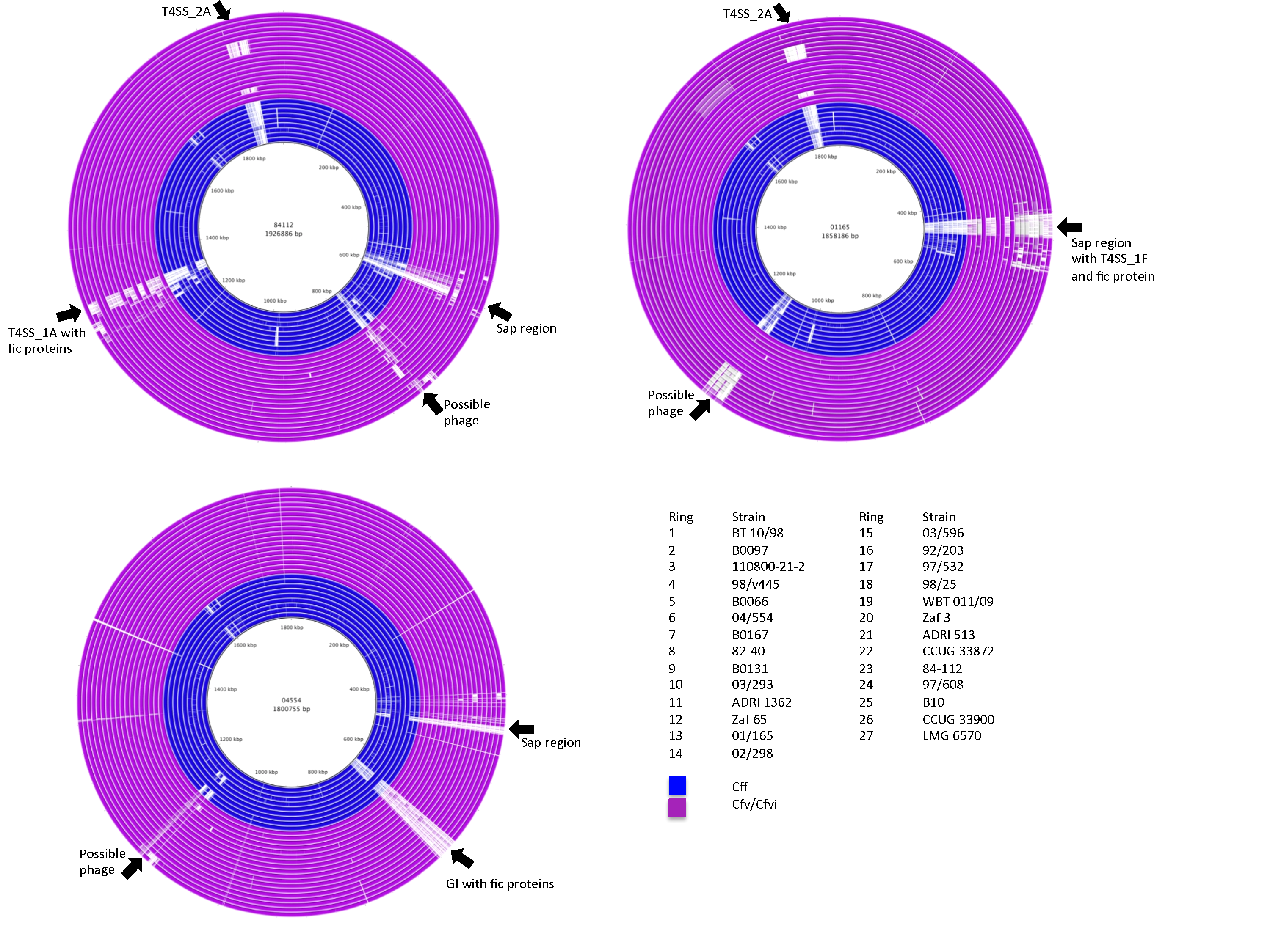

Supplement: S2 Fig — Shown are the locations of the chromosomal T4SS regions 1A, 1E and 2A in the reference genomes, using strain 84–112 or strain 01/165 as reference. Strain 04/554 is used as reference to show the location of a genomic island with four fic-encoding sequences. (TIF) [file pone.0152832.s002.tif]
